# Interleukin-15 Affects Patient Survival through Natural Killer Cell Recovery after Autologous Hematopoietic Stem Cell Transplantation for Non-Hodgkin Lymphomas

**DOI:** 10.1155/2010/914945

**Published:** 2010-04-18

**Authors:** Luis F. Porrata, David J. Inwards, Ivana N. Micallef, Patrick B. Johnston, Stephen M. Ansell, William J. Hogan, Svetomir N. Markovic

**Affiliations:** ^1^Division of Hematology, Department of Medicine and Blood and Marrow Program, Mayo Clinic, 200 First Street SW, Rochester, MN 55905, USA; ^2^Division of Hematology, Oncology Department, Mayo Clinic, 200 First Street SW, Rochester MN 55905, USA

## Abstract

Natural killer cells at day 15 (NK-15), after autologous peripheral blood hematopoietic stem cell transplantation (APHSCT), is a prognostic factor for overall survival (OS) and progression-free survival (PFS) in non-Hodgkin lymphoma (NHL). The potential role of the immunologic (homeostatic) environment affecting NK-15 recovery and survival post-APHSCT has not been fully studied. Therefore, we evaluate prospectively the cytokine profile in 50 NHL patients treated with APHSCT. Patients with an interleukin-15 (IL-15) ≥ 76.5 pg/mL at day 15 post-APHSCT experienced superior OS and PFS compared with those who did not; median OS; not reached versus 19.2 months, *P* < .002; and median PFS; not reached versus 6.8 months, *P* < .002, respectively. IL-15 was found to correlate with (*r*_*s*_ = 0.7, *P* < .0001) NK-15. Multivariate analysis showed only NK-15 as a prognostic factor for survival, suggesting that the survival benefit observed by IL-15 is most likely mediated by enhanced NK cell recovery post-APHSCT.

## 1. Introduction

Day 15 absolute lymphocyte count (ALC-15) after autologous peripheral blood hematopoietic stem cell transplantation (APHSCT) has been shown to be a significant predictor for survival in multiple hematologic malignancies [1–9] as well as solid tumors [10–12]. Natural killer cells at day 15 (NK-15) have been identified as the key lymphocyte subset in the ALC-15 that has a direct impact on survival post-APHSCT [13]. In addition, the lymphocytes collected at the same time with stem cells and infused to the patients have a direct impact on ALC-15 and NK-15 post-APHSCT, suggesting that the autograft can be viewed not only as the means to achieve hematologic engraftment by the infusion of stem cells but also as an adoptive immunotherapeutic maneuver by infusion of an autograft absolute lymphocyte count (A-ALC) affecting immune recovery and survival post-APHSCT [14–16]. Adoptive cellular therapy depends on the ability to optimally select the desired antigenic specificity immune effector cells and then induce cellular proliferation while preserving the effector function, engraftment, and homing abilities of the lymphocytes [17]. The infusion of A-ALC will provide the desired antigenic immune effector cells, but what factors to induce cellular proliferation while preserving the effector function, engraftment and homing abilities of the lymphocytes to provide an effective adoptive cellular therapy in APHSCT are currently not well defined. Therefore, we set out to investigate the cytokine milieu at day 15 post-APHSCT to assess which cytokines affect NK-15 and the superior survival mediated by a faster NK-15 recovery post-APHSCT. To achieve this goal the following endpoints were evaluated in the study: (1) to assess a correlation between cytokines and NK-15 post-APHSCT, (2) to assess if interleukin-15 (IL-15) affects survival post-APHSCT and through which immune effector cell, and (3) to identify a source of IL-15 production at day 15 post-APHSCT.

## 2. Material and Methods

### 2.1. Patient Population

Patients in the study were the same patient group enrolled in prospective study from February 2002 to February 2007 to assess the role of ALC-15 and NK-15 on survival post-APHSCT [13]. For this study 27 normal controls donated blood samples to assess cytokines levels. All patients signed written, informed consent to participate in the study. Approval of the study was obtained from the Mayo Clinic institutional review board and was in accordance with U.S. federal regulations and the Declaration of Helsinki.

### 2.2. A-ALC, ALC-15, Monocytes, and NK-15

Autograft absolute lymphocyte count (A-ALC) was determined as previously reported: % collection lymphocytes × absolute autograft white blood cell count/kilogram (kg) [18]. Autograft monocyte count (A-mono) was determined as follows: % collection monocytes × absolute autograft white blood cell count/kg. ALC-15 and monocytes at day 15 (mono-15) were determined from the differential white blood cell count obtained at day 15 post-APHSCT. NK-15, defined as CD16^+^/CD56^+^/CD3^−^, was according to manufacture's recommendations. Stained cells were then analyzed using flow cytometry (FACS callibur, Becton-Dickson, CA). Cells were analyzed for the percentage of cells expressing said antigens as well as average quantity of antigen expression [13].

### 2.3. Cytokine Assay

Cytokines were measured using the Bio-rad (Hercules, CA) human 27-plex system. Briefly, diluted human plasma was incubated for 30′ at room temperature with washed beads. The beads are coated with antibodies to the cytokines of interest. After incubation with patient plasma the beads were washed and incubated for 30′ with biotinylated 20 antibodies, followed by incubation with PE-conjugated streptavidin. Quantization of cytokines was performed on the Luminex 200 system (Austin, TX). The cytokines tested included Interleukin (IL)-1b, IL-1ra, IL-2, IL-4, IL-5, IL-6, IL-7, IL-8, IL-9, IL-10, IL-12p70, IL-13, IL-15, IL-17, Eotaxin, fibroblast growth factor (FGF), granulocyte colony-stimulating factor (G-CSF), granulocyte-macrophage colony-stimulating factor (GM-CSF), interferon-gamma (IFN-G), interferon-gamma-inducible protein 10 (IP-10), monocyte chemoattractant protein-1 (MCP-1), macrophage inflammatory proteins 1a and 1b (MIP-1a and MIP-1b), platelet-derived growth factor (PDGF), RANTES, tumor-necrosis-factor-gamma (TNF-G), and vascular endothelial growth factor (VEGF).

### 2.4. Prognostic Factors

Prognostic factors for post-APHSCT OS and PFS evaluated in this study included cytokines, ALC-15, NK-15, mono-15, international prognostic index (IPI) factors, infused CD34, and disease status at transplant.

### 2.5. Peripheral Blood Stem Cell Collection

All patients received G-CSF (10 *μ*g/kg) daily for 5 to 7 consecutive days by subcutaneous injection. Once their peripheral blood CD34^+^ cell count was ≥10 cells/*μ*L, patients began daily apheresis until they achieved a target of 5 × 10^6^ CD34^+^ cells/kg. A minimum target of 2 × 10^6^ CD34^+^ cells/kg was required for the patient to be considered for transplantation. Patients were assigned to the Baxter Amicus (Baxter, healthcare Corp., Deerfield, IL), Fenwal CS 3000 Plus (Baxter) or COBE Spectra (Gambro BCT, Lakewood, CO) based on instrument availability on the day of collection. Instrument setting used for the collection procedures has previously been described (ref). The number of apheresis collections was dependent upon collection of an adequate number of CD34^+^ cells to achieve hematologic engraftment post-APHSCT.

### 2.6. Conditioning Regimen

All patients received Carmustine (BCNU) 300 mg/m^2^ on day-6; etoposide 100 mg · m^2^ twice a day on days-5, -4, -3, and -2; and melphalan 140 mg/m^2^ on day-1 (BEAM). All patients received growth factor support starting on day +6 with sargramostim 500 *μ*g daily until evidence of neutrophilic engraftment defined as an absolute neutrophil count (ANC) ≥ 500 cells/*μ*g on 3 consecutive days.

### 2.7. Response and Survival

Response criteria were based on the guidelines from the non-Hodgkin lymphoma (NHL) international Workshop [19]. OS was measured from the date of transplant to the date of death, or last follow-up. PFS was defined as the time from transplant to the time of progression, relapse, death, or last follow-up.

### 2.8. Statistical Analysis

OS and PFS were analyzed using the approach of Kaplan and Meier [20]. Differences between survival curves were tested for statistical significance using the 2-tailed log-rank test. The Cox proportional hazard model [21] was used for the univariate and multivariate analysis to evaluate cytokines, NK-15, ALC-15, and mono-15 as a prognostic factor for post-APHSCT OS and PFS times.

In addition to the evaluation of cytokines (specifically IL-15) and its prognostic significance for OS and PFS, its utility as a marker of NK-15 was also assessed. The choice of optimal cutoff of IL-15 was based on its utility as a marker of NK-15 using Receiver operating characteristics (ROCs) curves and area under the curve (AUC) as well as its prognostic value for post-APHSCT OS and PFS. Prediction of NK-15 was explored further in logistic regression models, univariately assessing continuous and dichotomized values of A-ALC, cytokines, and mono-15. *χ* ^2^-tests were used to determine relationships between categorical variables. The Wilcoxon-rank test was used to determine associations between continuous variable and categories, and Spearman correlation coefficients were used to evaluate associations for continuous variables. Owing to multiple factors tested in the univariate analysis, multiple comparison corrections using Bonferroni correction procedure were applied declaring statistical significance at *P* < .002 (*α* = 0.05/*n*, *n* = 27 cytokines). 

The cut-off of ALC-15 ≥ 500 cells/*μ*L, A-ALC ≥ 0.5 × 10^9^ lymphocytes/kg, and NK-15 ≥ 80 cells/*μ*L was based on data from our previous studies [13–15].

## 3. Results

### 3.1. Patient Characteristics

The median age at the time of transplant for this cohort of 50 NHL patients was 57.7 years (range: 23–73). Baseline characteristics for the patients are described on Table 1. No patients were lost to follow-up. The updated median follow-up for the cohort was 25.2 months (range: 6–80.8 months). By the time of this analysis 25 patients (50%) had evidence of relapse and 18 patients (36%) had died. The transplant-related mortality for the cohort was only 2% (1 of 50). One patient died of an intracranial bleed, the rest because of progressive lymphoma. No patient received fludarabine-based regimens prior to APHSCT.

Table 2 shows the cytokine levels comparison between normal volunteers and the cohort of patients before APHSCT and at day 15 post-APHSCT. The following cytokines showed increased levels at day 15 post-APHSCT compared with normal volunteers and before APHSCT: IL-5, IL-6, IL-8, IL-10, IL-15, IP-10, MCP-1, and MIP-1b. No sargramostim was administered on day 15 post-APHSCT during research blood collection for the study as all patients engrafted their neutrophils prior to day 15 post-APHSCT.

### 3.2. IL-15 and NK-15

**Figure 1:**
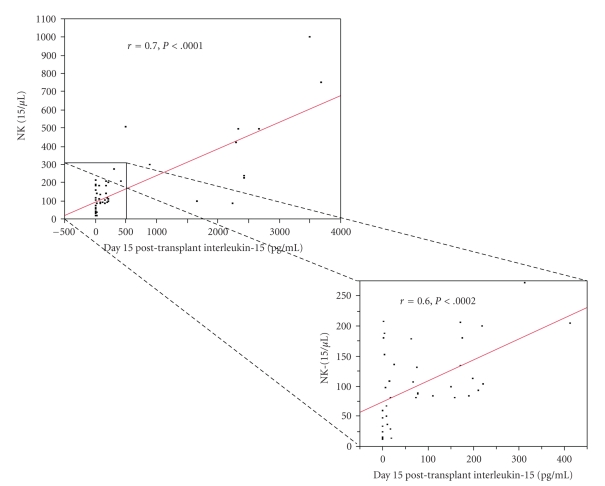
Scatterplot comparing IL-15 and NK-15 at day 15 post-APHSCT. Strong positive correlation was identified between IL-15 and NK-15 post-APHSCT (Spearman correlation rho factor, *r*_*s*_ = 0.7, *P* < .0001). Only 11 patients had IL-15 level higher than 500 pg/mL. To rule out the possibility that these 11 patients made skewed the data resulting in the strong correlation between IL-15 and NK-15, we truncated and analyzed the data for the 39 patients that had IL-15 levels between 0 and 500 pg/mL. A strong positive correlation was still observed between IL-15 and NK-15 (Spearman correlation rho factor, *r*_*s*_ = 0.6, *P* < .0002).

**Figure 2:**
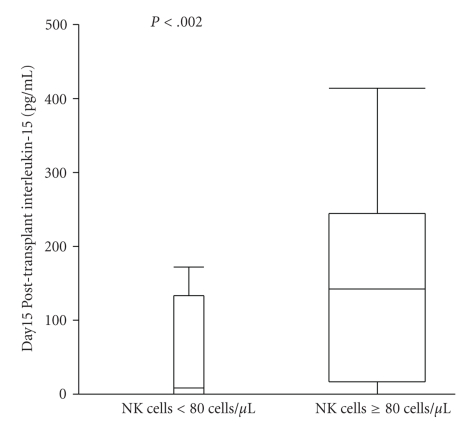
Box plot comparing the IL-15 levels at day 15 post-APHSCT between patients with an NK-15 ≥ 80 cells/*μ*L and NK-15 < 80 cells/*μ*L. Patients with a NK-15 ≥ 80 cells/*μ*L had a higher IL-15 levels (median of 155 pg/mL) compared with patients with aNK-15 < 80 cells/*μ*L (median of 8.89 pg/mL) (*P* < .002) at day 15 post-APHSCT.

We previously reported that NK-15 is the key lymphocyte subset at day 15 affecting survival post-APHSCT. Thus we set out to investigate which cytokine affects NK-15 recovery post-APHSCT. Table 3 shows the cytokine univariate analysis for correlation with NK-15. IL-15 and fibroblast growth factor (FGF) were strongly correlated with NK-15. We also published that A-ALC affected NK-15 recovery. In logistic regression analysis, both A-ALC (*P* < .001) and IL-15 (*P* < .002) retained their ability to affect NK-15 recovery post-APHSCT. IL-15 was found to be both a strong predictor for area under the curve (AUC = 0.87, *P* < .002) and strongly correlated with (*r*_*s*_ = 0.7, *P* < .0001) NK-15. Figure 1 shows the scatter plot for IL-15 and NK-15. Only 11 patients had IL-15 level higher than 500 pg/mL. To rule out the possibility that these 11 patients made skewed the data resulting in the strong correlation between IL-15 and NK-15, we truncated and analyzed the data for the 39 patients that had IL-15 levels between 0 and 500 pg/mL. We still observed a strong correlation between IL-15 and NK-15 (*r*_*s*_ = 0.6, *P* < .0002). If we dichotomized the NK-15, patients with an NK-15 ≥ 80 cells/*μ*L had higher levels of IL-15 compared with those who did not (median IL-15 of 155 pg/mL versus 8.89 pg/mL, *P* < .002) (Figure 2).

### 3.3. Cytokines and Survival Post-APHSCT

Table 4 shows the cytokines univariate analysis for survival post-APHSCT. The cytokines were evaluated as continuous variables. IL-15 at day 15 post-APHSCT was the only cytokine that was statistically significant for OS. IL-15 and PDGF were statistically significant for PFS. In the multivariate analysis for PFS, IL-15 was the only cytokine that remained statistically significant (IL-15: HR = 0.950; 95%  CI = 0.824–0.978, *P* < .002; PDGF: HR = 1.020; 95%  CI = 1.010–1.058, *P* = .02).

**Figure 3:**
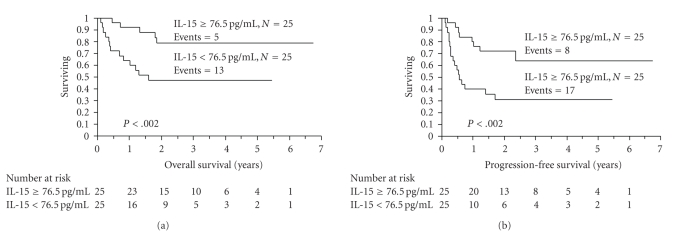
(a) Overall survival (OS) of patients with an interleukin-15 (IL-15) ≥ 76.5 pg/mL versus patients with an IL-15 < 76.5 pg/mL. The median OS was not reached in the group of patients with an IL-15 ≥ 76.5 pg/mL and 19.2 months with the group of patients with an IL-15, 76.5 pg/mL. The OS rates at 3 years were 79% and 47%, respectively (*P* < .002). (b) Progression-free survival (PFS) of patients with an interleukin-15 (IL-15) ≥ 76.5 pg/mL versus patients with an IL-15 < 76.5 pg/mL. The median PFS was not reached in the group of patients with an IL-15 ≥ 76.5 pg/mL and 6.8 months with the group of patients with an IL-15, 76.5 pg/mL. The PFS rates at 3 years were 64% and 31%, respectively (*P* < .002).

A cut-off of 76.5 pg/mL for IL-15 was supported by being the median value of the data as well as it yielded the greatest differential in survival based on *χ* ^2^ values analyzed at different cutoff points (between the 25th and 75th quartiles) from the log-rank tests. Patients with an IL-15 ≥ 76.5 pg/mL (*n* = 25) experienced superior OS (Figure 3(a)) and PFS (Figure 3(b)) compared with those who did not (*n* = 25); median OS; not reached versus 19.2 months, 3 years OS rates of 79% versus 47%, *P* < .002; and median PFS; not reached versus 6.8 months, 3 years PFS rates of 64% versus 31%, *P* < .002, respectively. 

In order to minimize uncontrolled factors that could have affected IL-15 levels post-APHSCT, we analyzed patients characteristics based on IL-15 ≥ 76.5 pg/mL versus an IL-15 < 76.5 pg/mL (Table 5). We also analyzed if the pre-transplant IL-15 levels were associated with day 15 post-APHSCT IL-15 levels. We found a positive correlation between pre- and post-APHSCT IL-15 levels (*r* = 0.6, *P* < .0001).

**Figure 4:**
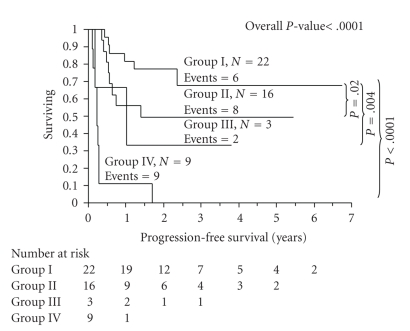
Progression-free survival based on categorized subset analysis of NK-15 and IL-15 levels. Group I was patients with an NK-15 ≥ 80 cells/*μ*L and IL-15 ≥ 76.5 pg/mL; group II was NK-15 ≥ 80 cells/*μ*L and IL-15 < 76.5 pg/mL; group III was NK-15 < 80 cells/*μ*L and IL-15 ≥ 76.5 pg/mL; group IV was NK-15 < 80 cells/*μ*L and IL-15 < 76.5 pg/mL. The 3-year PFS rate for group I was 68%; group II was 49%; group III was 33%; group IV was 0%.

To assess the role on survival of NK-15 and IL-15, we categorized the patients into 4 groups: group I was patients with an NK-15 ≥ 80 cells/*μ*L and IL-15 ≥ 76.5 pg/mL; group II was NK-15 ≥ 80 cells/*μ*L and IL-15 < 76.5 pg/mL; group III was NK-15 < 80 cells/*μ*L and IL-15 ≥ 76.5 pg/mL; group IV was NK-15 < 80 cells/*μ*L and IL-15 < 76.5 pg/mL. We observed worsening PFS in patients with progressing low NK-15 and IL-15 levels compared with patients with high NK-15 and IL-15 levels (3 years PFS rates for group I was 68%; group II was 49%; group III was 33%; group IV was 0%) (Figure 4). In the multivariate analysis comparing NK-15 and IL-15 for OS and PFS, NK-15 truncated IL-15 (OS; HR = 0.32, *P* = .02; and PFS: HR = 0.33, *P* = .01).

#### 3.3.1. Source of IL-15 at Day 15 Post-APHSCT (Monocytes/Macrophages)

**Figure 5:**
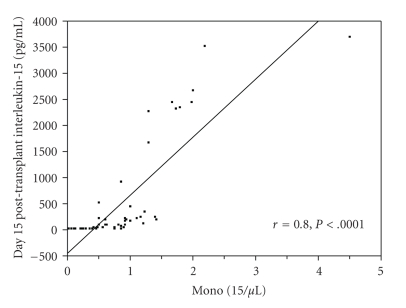
Scatterplot comparing IL-15 and mono-15 at day 15 post-APHSCT. Strong positive correlation was identified between IL-15 and mono-15 post-APHSCT (Spearman correlation rho factor, *r*_*s*_ = 0.8, *P* < .0001).

**Figure 6:**
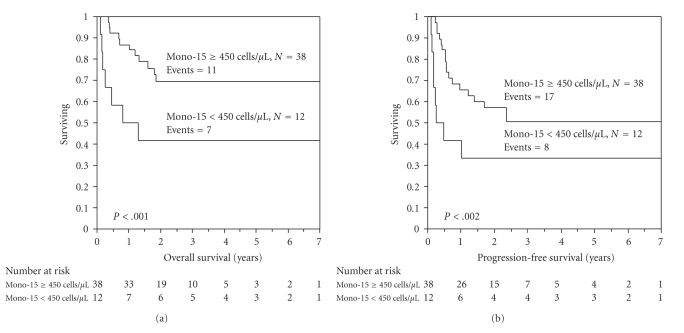
(a) Overall survival (OS) of patients with a mono-15 ≥ 450 cells/*μ*L versus patients with a mono-15 < 450 cells/*μ*L. The median OS was not reached in the group of patients with a mono-15 ≥ 450 cells/*μ*L and 12.6 months with the group of patients with a mono-15 < 450 cells/*μ*L. The OS rates at 3 years were 70% and 42%, respectively (*P* < .001). (b) Progression-free survival (PFS) of patients with a mono-15 ≥ 450 cells/*μ*L versus patients with a mono-15 < 450 cells/*μ*L. The median PFS was not reached in the group of patients with a mono-15 ≥ 450 cells/*μ*L and 4.5 months with the group of patients with a mono-15 < 450 cells/*μ*L. The PFS rates at 3 years were 71% and 33%, respectively (*P* < .002).

The monocytic/macrophage system has been reported as a source of IL-15. Thus, we set out to investigate that the monocytic count at day 15 (mono-15) post-APHSCT affects IL-15 levels. We identified a good correlation between IL-15 and mono-15 (*r*_*s*_ = 0.8, *P* < .0001) (Figure 5). We also identified a good correlation between NK-15 and mono-15 (*r*_*s*_ = 0.54, *P* < .0001). However, in logistic regression analysis, IL-15 and A-ALC remained statistical significant as sources of NK-15 and truncated mono-15 (*P* = .1) (Table 6), suggesting that the mono-15 ability to affect NK-15 is mediated by the production of IL-15. Mono-15 was also associated with OS (*P* < .001) and PFS (*P* < .002) (Figures 6(a) and 6(b)). However, in multivariate analysis, NK-15 remained the only statical prognostic factor for survival compared with mono-15 (OS, *P* = .9; PFS, *P* = .9), or ALC-15 (OS, *P* = .8; PFS, *P* = .2) (Table 7). To assess the source of mono-15 recovery, we analyzed the number of A-mono infused and found a good correlation between A-mono and mono-15 (*r*_*s*_ = 0.5, *P* < .001). In addition, we identified a positive correlation between A-mono and NK-15 (*r* = 0.4, *P* < .008) and between A-mono and IL-15 at day 15 post-APHSCT (*r* = 0.3, *P* < .04).

## 4. Discussion

ALC-15 ≥ 500 cells/*μ*L is a prognostic factor for survival post-APHSCT. NK-15 is the key lymphocyte subset of ALC-15 contributing to the superior clinical outcomes that observed post-APHSCT. ALC-15 and NK-15 recovery depends on the infused A-ALC, suggesting the new concept that the autograft used to infuse stem cells in APHSCT can be used also as an adoptive immunotherapeutic maneuver to enhance immune recovery, translating into better clinical outcomes post-APHSCT. However, for an adoptive cellular therapy to be effective, it requires cellular proliferation while preserving the effector function. We have previously published that NK cells at two weeks post-APHSCT are functionally active [22]. Thus, we studied the cytokine milieu at day 15 post-APHSCT to assess its interaction with NK-15 recovery and survival post-APHSCT.

Our cytokine profile at day 15 post-APHSCT is similar to what has been previously reported in autologous stem cell transplantation. [23, 24]. However, to our knowledge this is the first study looking at the role of IL-15 on NK-15 recovery and survival post-APHSCT. IL-15 was the only cytokine at day 15 post-APHSCT that was associated with survival post-transplant. IL-15 has been reported to support the homeostasis of IL-2/IL-15R*β* expressing memory CD8+ T cells and NK cells via transpresentation of IL-15 by IL-15R*α* expressing cells [25, 26]. We previously reported that NK-15 is the key lymphocyte subset of ALC-15 affecting survival post-APHSCT [13]. Since IL-15 has been associated with NK cells homeostasis and differentiation, we set out to identify if there was an association between IL-15 and NK-15 post-APHSCT. We identified a strong correlation between IL-15 and NK-15 as a continuous or dichotomized variable. Our finding is similar to what has been reported in allogeneic stem cell transplantation, where faster NK cell recovery early post allogeneic stem cell transplantation is associated with elevated IL-15 levels [27]. Despite the small number of patients, patients with higher NK-15 and IL-15 levels experienced better PFS compared with patients with progressing lower NK-15 and IL-15 levels. Multivariate analysis showed that NK-15 truncated IL-15 levels, suggesting that the possibility that the survival benefit observed by IL-15 is mediated by enhanced NK cell recovery post-APHSCT. Another factor associated with NK-15 and previously reported was A-ALC. A-ALC was an independent factor affecting NK-15 recovery when compared with IL-15, suggesting that A-ALC, as an adoptive cellular therapy, provides a direct influx of immune effector cells (i.e., NK cells), while IL-15 helps to sustain the proliferation of NK cells.

Due to the apparent role of IL-15 on NK-15 recovery, we set out to investigate possible sources of IL-15 at day 15 post-APHSCT. Monocytes have been implicated as a source of IL-15 [28]. Thus, we studied the monocyte recovery at day 15 (mono-15) post-APHSCT, as a surrogate marker of IL-15 production, and its correlation with IL-15 levels. We identified a strong correlation between mono-15 and IL-15 at day 15 post-APHSCT. We also found an association between mono-15 and NK-15. However, a multivariate analysis showed IL-15 and not mono-15 as the factor affecting NK-15 recovery, suggesting that the effect on NK-15 recovery by mono-15 is most likely mediated by the production of IL-15 by mono-15. Another possible source of IL-15 is from dendritic cells [28]. Recently, it has been reported that the infusion of autograft dendritic cells as well as their recovery post transplant affects survival in lymphoma patients treated with APHSCT [29]. It is reasonable to hypothesize that the survival benefit from the infusion and recovery of dendritic cells post-APHSCT could also be related due to the production of IL-15 by dendritic cells activating NK cells*. *However, it is important to mention other factors that could affect the IL-15 levels during the peri-transplant period including (1) depletion of the lymphoid populations that normally consumed circulating IL-15 due to the conditioning regimens [27], or (2) response to the inflammatory environment triggered by the conditioning regimens [30, 31].

A limitation of the study is that even though we identified prospectively a correlation between IL-15 and NK-15 leading to better survival post-APHSCT, it does not prove causation. This limitation is based on the fact that this is a prospective correlative study which might not have controlled for factors that could have affect IL-15 levels post-APHSCT such as (1) prior treatments before APHSCT and fludarabine-based regimens that could have affected A-ALC collection, ALC-15 recovery (i.e., NK-15), as well as mono-15 recovery post-APHSCT; and (2) the use of sargramostim affecting cytokine production. However, patient's baseline characteristics were overall balanced regardless if IL-15 levels were equal/above or below 76.5 pg/mL. The positive correlation between pre- and post-APHSCT IL-15 levels suggests that the pretransplant IL-15 levels might help to anticipate prognosis after APHSCT. We hope that our ongoing Phase III randomized double-blind trial assessing if higher infusion of A-ALC translates into faster immune recovery and thus better survival post-APHSCT will address the relationship between IL-15 and NK cells as ancillary immunologic studies will be performed on this trial. In this trial none of the patients will receive sargramostim post-APHSCT. The premise of this phase III study is based on the fact that we have shown that by manipulating the mononuclear cell band during the centrifugation process of the apheresis machine, we can collect higher numbers of naïve T-cells and NK cells with direct impact on immune recovery post-APHSCT [32]. In this study, patients will be randomized to collect stem cells with the standard apheresis machine settings versus the modified apheresis machine settings, aiming at collecting more immune effector cells. Similarly, the results of the current study suggest that enriching the autograft with monocytes might also have a direct impact on NK-15 recovery and survival post-APHSCT. This hypothesis will be tested in the phase III study.

**Figure 7:**
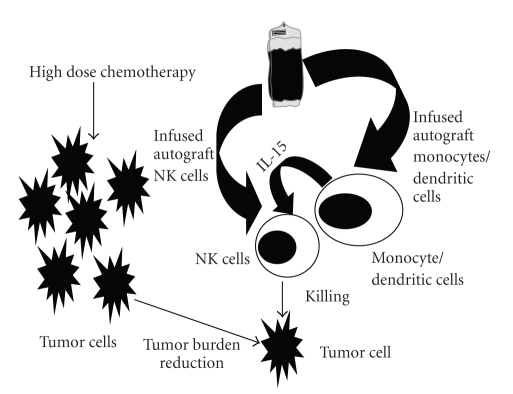
Schematic representation of autologous graft-versus-tumor effect (hypothesis).

Based on the results of this study it appears that NK-15 recovery (the key lymphocyte subset of ALC-15 affecting survival post-APHSCT) depends on the adoptive infusion from A-ALC and the homeostatic/proliferative effect of IL-15. Monocytes/dendritic cells appear to mediate an NK cell supportive “homeostatic environment” potentially facilitating NK cells engraftment mediated by IL-15 following-APHSCT, ultimately leading to a therapeutic autologous graft-versus-tumor effect (Figure 7). Evaluation of this hypothesis is currently underway on our Phase III study (Study ID: CDR0000577897, MAYO-MC0681).

In summary, this is the first study showing the role IL-15 on survival post-APHSCT most likely by supporting NK cells engraftment. In addition, this study also shows monocytes as possible source of IL-15, suggesting that the monocyte count can be used as a surrogate marker to assess the cytokine milieu (specifically IL-15) post-APHSCT. Further studies are warranted to understand the role of cytokines on survival post-APHSCT to develop more effective immunotherapeutic treatments in the APHSCT setting.

## Figures and Tables

**Table 1:** Baseline characteristics of the cohort.

Characteristics	*n* (%)	Median	Range
Age at transplant, years	50 (100%)	57.5	(23–73)
Sex			
Female	17 (34%)		
Male	33 (66%)		
Lymphoma type			
DLBCL	34 (68%)		
Mantle cell	10 (20%)		
T-cell	5 (10%)		
Follicular	1 (2%)		

*Prognostic factors at diagnosis*			
Extra nodal sites			
0	33 (66%)		
1	15 (30%)		
2	2 (4%)		
LDH (U/L)	50 (100%)	209	(150–1515)
Performance status			
0	31 (62%)		
1	17 (34%)		
2	2 (4%)		
Stage			
I	6 (12%)		
II	5 (10%)		
III	8 (16%)		
IV	31 (62%)		

*IPI score*			
Age at transplant, years			
≥60	23 (46%)		
<60	27 (56%)		
LDH			
Normal	21 (42%)		
Abnormal	29 (58%)		
Extra nodal sites			
≥2	2 (4%)		
<2	48 (96%)		
Performance status			
≥2	2 (4%)		
<2	48 (96%)		
Stage			
I/II	11 (22%)		
III/IV	39 (78%)		
Infused CD34 cells × 10^6^/kg	50 (100%)	4.68	(2.03–8.56)
Infused A-ALC × 10^9^ lymphocytes/kg	50 (100%)	0.55	(0.08–1.5)
ALC-15 cells/*μ*L	50 (100%)	670	(2.0–4,500)
NK-15 cells/*μ*L	50 (100%)	103	(10–999)
Mono-15 cells/*μ*L	50 (100%)	860	(2.0–4,500)
Infused A-mono × 10^9^ monocytes/kg	50 (100%)	0.58	(0.08–1.5)

A-ALC: autograft, absolute lymphocyte count; ALC-15: absolute lymphocyte count at day 15 post transplant; A-mono: autograft absolute monocyte count; DLBCL: diffuse large B-cell lymphoma; IPI: International prognostic index; LDH: lactate dehydrogenase; Mono-15: absolute monocyte count at day 15 post transplant; NK-15: absolute natural killer cell count at day 15 post transplant.

**Table 2:** Cytokines levels comparison between normal volunteers, before transplantation, and at day 15 after transplantation.

Cytokines	Normal	Pretransplant	Day 15 post transplant	*P*-value Normal versus Pretransplant	*P*-value Normal versus Day 15 post transplant	*P*-value Pre versus Day 15 post transplant
	Median (25th-75th quartiles)	Median (25th-75th quartiles)	Median (25th-75th quartiles)			
IL-1b	3.59 (2.32–5.55)	4.72 (3.48–7.79)	2.99 (1.3–8.11)	.5	.8	.1
IL-1ra	120.12 (90.59–287.77)	356.56 (228.95–662.87)	283.33 (142.52–434.73)	<.0001	<.0001	.01
IL-2	0 (0–56.12)	4.24 (0–23.87)	0 (0–7.89)	.01	.7	<.0004
IL-4	3.09 (2.09–3.99)	3.95 (3.33–5.43)	2.12 (1.25–3.63)	.6	.7	.02
IL-5	0.92 (0.69–1.67)	2.82 (1.79–3.96)	4.3 (2.93–13.75)	<.0001	<.0001	.01
IL-6	0 (0–2.14)	11.78 (5.6–21.05)	24.48 (9.84–48.72)	<.0001	<.0001	.01
IL-7	24.64 (13.59–39.08)	21.99 (16.85–39.95)	15.66 (5.77–26.61)	.04	.007	.02
IL-8	2.57 (0.35–7.71)	30.89 (19.91–97.72)	36.67 (27.02–71.78)	<.0001	<.0001	.2
IL-9	4.09 (0–68.28)	40.18 (4.93–91.22)	27.58 (8.29–64)	.04	.04	.003
IL-10	1.97 (1.24–3.84)	4.91 (3.91–8.16)	8.94 (5.91–13.61)	.01	<.0001	<.002
IL-12p70	6.7 (2.52–16.35)	15.27 (7.29–29.68)	7.61 (4.45–12.93)	<.0006	.2	<.0004
IL-13	2.93 (1.33–4.17)	2.49 (1.71–5.26)	1.46 (0.56–2.31)	.02	.5	<.002
IL-15	0 (0–0)	8.75 (0.07–110.5)	76.5 (5.4–219.22)	<.0001	<.0001	<.0001
IL-17	10.62 (0–30.66)	18.43 (4.33–49.92)	7.08 (0–17.14)	.01	.2	<.0001
Eotaxin	49.39 (41.43–76.41)	68.18 (42.26–104.04)	73.76 (44.96–108.87)	.02	<.0001	.6
FGF	0 (0–0)	0 (0–84.64)	0 (0–13.07)	.7	.8	.7
G-CSF	989 (385–1243)	83.08 (60.01–124.8)	67.48 (45.74–93.55)	<.0001	<.0001	<.0001
GM-CSF	161.74 (32.26–1142.8)	80.99 (41.13–173.59)	63.75 (25.4–164.6)	.01	<.0001	<.002
IFN-G	142.49 (87.71–264.05)	125.06 (78.04–193.52)	65.65 (28.81–86.42)	.03	<.0009	<.0001
IP-10	245.22 (150.9–326.4)	915.6 (585.98–1783.83)	1440.53 (102.2–3067.40)	<.0001	<.0001	<.0001
MCP-1	41.3 (0–90.25)	86.34 (33.83–146.48)	120.43 (54.46–209.55)	.02	<.0001	.008
MIP-1a	7.4 (4.58–10.52)	12.9 (8.62–31.7)	8.56 (5.35–1363)	<.001	.7	<.001
MIP-1b	51.23 (21.15–106.98)	97.36 (57.01–256.38)	104.34 (63.59–179.76)	<.002	<.0001	.6
PDGF	1260.24 (805.22–2289.82)	2071.16 (1060.34–3485.72)	423.71 (112.94–697.84)	<.0001	<.0001	<.0001
RANTES	2650.98 (1584.55–4500)	2600 (2600–3000)	2302.37 (297.92–1072.83)	.7	.1	<.001
TNF-G	0.74 (0–188.99)	70.48 (52.82–132.48)	32.06 (23.37–82.85)	.02	.07	<.0001
VEGF	23.13 (12–55.42)	77.38 (28.42–104.34)	36.52 (10.28–91.85)	<.0007	<.002	<.0001

Interleukin (IL)-1b, IL-1ra, IL-2, IL-4, IL-5, IL-6, IL-7, IL-8, IL-9, IL-10, IL-12p70, IL-13, IL-15, IL-17, Eotaxin, fibroblast growth factor (FGF), granulocyte colony-stimulating factor (G-CSF), granulocyte-macrophage colony-stimulating factor (GM-CSF), interferon-gamma (IFN-G), interferon-gamma-inducible protein 10 (IP-10), monocyte chemoattractant protein-1 (MCP-1), macrophage inflammatory proteins 1a and 1b (MIP-1a and MIP-1b), platelet-derived growth factor (PDGF), RANTES, tumor-necrosis-factor-gamma (TNF-G), and vascular endothelial growth factor (VEGF).

**Table 3:** Univariate analysis of the correlation between cytokines and NK-15.

Cytokines	Spearman (rho) correlation	*P*-value
IL-1b	0.17	.2
IL-1ra	0.09	.5
IL-2	0.06	.7
IL-4	0.2	.1
IL-5	0.2	.2
IL-6	0.1	.3
IL-7	0.06	.6
IL-8	0.2	.1
IL-9	0.2	.1
IL-10	−0.2	.3
IL-12p70	0.1	.4
IL-13	0.1	.5
IL-15	0.7	<.0001
IL-17	0.1	.4
Eotaxin	0.1	.3
FGF	0.6	<.0001
G-GSF	0.1	.3
GM-CSF	0.1	.4
IFN-G	0.2	.2
IP-10	0.1	.4
MCP-1	0.06	.7
MIP-1a	0.2	.2
MIP-1b	0.2	.2
PDGF	0.2	.1
RANTES	0.06	.7
TNF-*α*	0.3	.06
VEGF	0.2	.2

Interleukin (IL)-1b, IL-1ra, IL-2, IL-4, IL-5, IL-6, IL-7, IL-8, IL-9, IL-10, IL-12p70, IL-13, IL-15, IL-17, Eotaxin, fibroblast growth factor (FGF), granulocyte colony-stimulating factor (G-CSF), granulocyte-macrophage colony-stimulating factor (GM-CSF), interferon-gamma (IFN-G), interferon-gamma-inducible protein 10 (IP-10), monocyte chemoattractant protein-1 (MCP-1), macrophage inflammatory proteins 1a and 1b (MIP-1a and MIP-1b), platelet-derived growth factor (PDGF), RANTES, tumor-necrosis-factor-gamma (TNF-G), and vascular endothelial growth factor (VEGF).

**Table 4:** Univariate analysis for overall and progression-free survival.

Cytokines	Overall survival *P*-value	Progression-free survival *P*-value
IL-1b	.08	.1
IL-1ra	.2	.4
IL-2	.09	.1
IL-4	.03	.02
IL-5	.8	.3
IL-6	.07	.08
IL-7	.06	.06
IL-8	.01	.04
IL-9	.1	.2
IL-10	.02	.04
IL-12p70	.06	.06
IL-13	.06	.06
IL-15	<.0001	<.001
IL-17	.09	.1
Eotaxin	.4	.02
FGF	.2	.3
G-CSF	.06	.06
GM-CSF	.06	.06
IFN-G	.04	.06
IP-10	.4	.5
MCP-1	.1	.2
MIP-1a	.008	.008
MIP-1b	.07	.1
PDGF	.004	<.002
RANTES	.6	.7
TNF-*α*	.007	.006
VEGF	.4	.3
Age ≥ 60	.16	.06
A-ALC	.01	.004
ALC-15	<.0004	<.0001
CD34	.3	.8
Disease status at transplant: complete remission	.11	.1
Extra nodal sites ≥ 2	.46	.8
LDH; abnormal	.3	.2
Mono-15	<.001	<.002
NK-15	<.0002	<.0002
PS > 1	.24	.81
Stage III/IV	.2	.28

Interleukin (IL)-1b, IL-1ra, IL-2, IL-4, IL-5, IL-6, IL-7, IL-8, IL-9, IL-10, IL-12p70, IL-13, IL-15, IL-17, Eotaxin, fibroblast growth factor (FGF), granulocyte colony-stimulating factor (G-CSF), granulocyte-macrophage colony-stimulating factor (GM-CSF), interferon-gamma (IFN-G), interferon-gamma-inducible protein 10 (IP-10), monocyte chemoattractant protein-1 (MCP-1), macrophage inflammatory proteins 1a and 1b (MIP-1a and MIP-1b), platelet-derived growth factor (PDGF), RANTES, tumor-necrosis-factor-gamma (TNF-G), vascular endothelial growth factor (VEGF), autograft absolute lymphocyte count (A-ALC), absolute lymphocyte count at day 15 post transplant (ALC-15), lactate dehydrogenase (LDH), absolute monocyte count at day 15 post transplant (Mono-15), natural killer cells at day 15 post transplant (NK-15), and performance status (PS).

**Table 5:** Patients baseline characteristics based on IL-15 ≥ 76.5 pg/mL versus IL-15 < 76.5 pg/mL.

Characteristics	IL-15 < 76.5 pg/mL (*n* = 25)	IL-15 ≥ 76.5 pg/mL (*n* = 25)	*P*-value
Age at transplant, years: median (range)	62 (44–73)	54 (23–71)	.02
Gender			.2
Female	6	11	
Male	19	14	
Lymphoma type			.7
DLBCL	17	17	
Mantle cell	5	5	
T-cell	3	2	
Follicular	0	1	

*Prognostic factors at diagnosis*			
Extranodal disease			.2
0	15	17	
1	8	7	
2	2	0	
LDH (U/L), median (range)	222 (150–550)	200 (170–1515)	.5
Performance status			.1
0	12	19	
1	12	5	
2	1	1	
Stage			.3
I	2	4	
II	1	4	
III	4	4	
IV	18	13	

*IPI score*			
Age at transplant, years			.1
≥60	15	8	
<60	10	17	
Extranodal sites			.5
≥2	2	0	
<2	23	25	
LDH (U/L)			.3
Abnormal	13	8	
Normal	12	17	
Performance status			.9
≥2	1	1	
<2	24	24	
Stage			.2
I/II	3	8	
III/IV	22	17	
IPI index			.03
0	1	4	
1	4	11	
2	15	7	
3	5	3	
Disease status prior to transplant			.5
CR	6	9	
PR	19	16	
Infused CD34 cells × 10^6^/kg; median (range)	5.17 (2.2–8.56)	4.4 (2.03–8.1)	.2
ALC-15 cells/*μ*L: median (range)	0.65 (0.02–1.91)	0.68 (0.1–4.5)	.2
ALC-15 cells/*μ*L			.2
≥500	15	20	
<500	10	5	
Infused A-ALC × 10^9^ lymphocytes/kg: median (range)	0.55 (0.02–1.66)	0.63 (0.14–1.66)	.1
Infused A-ALC			.2
≥0.5 × 10^9^ lymphocytes/kg	14	19	
<0.5 ×10^9^ lymphocytes/kg	11	6	
Infused A-mono × 10^9^ monocytes/kg: median (range)	0.57 (0.06–1.5)	0.65 (0.26–1.5)	.3
Mono-15 cells/*μ*L: median (range)	0.85 (0.02–2)	0.86 (0.06–4.5)	.8
NK-15 cells/*μ*L: median (range)	98 (10–744)	130 (12–999)	.8

A-ALC: autograft, absolute lymphocyte count; ALC-15: absolute lymphocyte count at day 15 post transplant; A-mono: autograft absolute monocyte count; CR: complete remission; DLBCL: diffuse large B-cell lymphoma; IPI: International prognostic index; LDH: lactate dehydrogenase; Mono-15: absolute monocyte count at day 15 post transplant; PR: partial remission; NK-15: absolute natural killer cell count at day 15 post transplant.

**Table 6:** Logistic regression analysis of factors affecting NK-15 recovery post-APHSCT.

Factors	Estimate	Standard Error	*χ* ^2^	*P*-value
A-ALC	20.6	79.9	18.2	<.0001
IL-15 at day 15 post-APHSCT	19.9	85.6	8.3	<.004
Mono-15	1.32	0.75	1.7	.1

A-ALC: autograft, absolute lymphocyte count; APHSCT: autologous peripheral blood hematopoietic stem cell transplantation; IL-15: interleukin-15; Mono-15: absolute monocyte count at day 15 post transplant; *χ* ^2^: chi square.

**Table 7:** Multivariate analysis for overall and progression-free survival.

Factors	Overall survival			Progression-free survival		
	RR	95% CI	*P*-value	RR	95% CI	*P*-value
ALC-15	0.656	0.128–2.657	.8	0.628	0.152–2.107	.2
Mono-15	1.125	0.316–3.317	.9	1.060	0.374–2.637	.9
NK-15	0.176	0.062–0.797	<.006	0.188	0.057–0.593	<.002

ALC-15: absolute lymphocyte count at day 15 post transplant; Mono-15: absolute monocyte count at day 15 post transplant; and NK-15: natural killer cells at day 15 post transplant.

## References

[1] Porrata LF, Gertz MA, Inwards DJ (2001). Early lymphocyte recovery predicts superior survival after autologous hematopoietic stem cell transplantation in multiple myeloma or non-Hodgkin lymphoma. *Blood*.

[2] Porrata LF, Inwards DJ, Micallef IN, Ansell SM, Geyer SM, Markovic SN (2002). Early lymphocyte recovery post-autologous haematopoietic stem cell transplantation is associated with better survival in Hodgkin’s disease. *British Journal of Haematology*.

[3] Porrata LF, Litzow MR, Tefferi A (2002). Early lymphocyte recovery is a predictive factor for prolonged survival after autologous hematopoietic stem cell transplantation for acute myelogenous leukemia. *Leukemia*.

[4] Joao C, Porrata LF, Inwards DJ (2006). Early lymphocyte recovery after autologous stem cell transplantation predicts superior survival in mantle-cell lymphoma. *Bone Marrow Transplantation*.

[5] Kim H, Sohn H-J, Kim S, Lee J-S, Kim W-K, Suh C (2006). Early lymphocyte recovery predicts longer survival after autologous peripheral blood stem cell transplantation in multiple myeloma. *Bone Marrow Transplantation*.

[6] Boulassel MR, Herr AL, Deb Edwardes MD (2006). Early lymphocyte recovery following autologous peripheral stem cell transplantation is associated with better survival in younger patients with lymphoproliferative disorders. *Hematology*.

[7] Kim H, Sohn H-J, Kim S-E (2004). Lymphocyte recovery as a positive predictor of prolonged survival after autologous peripheral blood stem cell transplantation in T-cell non-Hodgkin’s lymphoma. *Bone Marrow Transplantation*.

[8] Gordan LN, Sugrue MW, Lynch JW, Williams KD, Khan SA, Moreb JS (2003). Correlation of early lymphocyte recovery and progression-free survival after autologous stem-cell transplant in patients with Hodgkin’s and non-Hodgkin’s lymphoma. *Bone Marrow Transplantation*.

[9] Porrata LF, Gertz MA, Litzow MR (2005). Early lymphocyte recovery predicts superior survival after autologous hematopoietic stem cell transplantation for patients with primary systemic amyloidosis. *Clinical Cancer Research*.

[10] Ferrandina G, Pierelli L, Perillo A (2003). Lymphocyte recovery in advanced ovarian cancer patients after high-dose chemotherapy and peripheral blood stem cell plus growth factor support: clinical implications. *Clinical Cancer Research*.

[11] Nieto Y, Shpall EJ, McNiece IK (2004). Prognostic analysis of early lymphocyte recovery in patients with advanced breast cancer receiving high-dose chemotherapy with an autologous hematopoietic progenitor cell transplant. *Clinical Cancer Research*.

[12] Porrata LF, Ingle JN, Litzow MR, Geyer S, Markovic SN (2001). Prolonged survival associated with early lymphocyte recovery after autologous hematopoietic stem cell transplantation for patients with metastatic breast cancer. *Bone Marrow Transplantation*.

[13] Porrata LF, Inwards DJ, Ansell SM (2008). Early lymphocyte recovery predicts superior survival after autologous stem cell transplantation in non-Hodgkin lymphoma: a prospective study. *Biology of Blood and Marrow Transplantation*.

[14] Porrata LF, Lotzow MR, Inwards DJ (2004). Infused peripheral blood autograft absolute lymphocyte count correlates with day 15 absolute lymphocyte count and clinical outcome after autologous peripheral hematopoietic stem cell transplantation in non-Hodgkin’s lymphoma. *Bone Marrow Transplantation*.

[15] Porrata LF, Gertz MA, Geyer SM (2004). The dose of infused lymphocytes in the autograft directly correlates with clinical outcome after autologous peripheral blood hematopoietic stem cell transplantation in multiple myeloma. *Leukemia*.

[16] Hiwase DK, Hiwase S, Bailey M, Bollard G, Schwarer AP (2008). Higher infused lymphocyte dose predicts higher lymphocyte recovery, which in turn, predicts superior overall survival following autologous hematopoietic stem cell transplantation for multiple myeloma. *Biology of Blood and Marrow Transplantation*.

[17] Aqui NA, June CH (2008). Post-transplant adoptive T-cell immunotherapy. *Best Practice and Research: Clinical Haematology*.

[18] Katipamula R, Porrata LF, Gastineau DA (2006). Apheresis instrument settings influence infused absolute lymphocyte count affecting survival following autologous peripheral hematopoietic stem cell transplantation in non-Hodgkin’s lymphoma: the need to optimize instrument setting and define a lymphocyte collection target. *Bone Marrow Transplantation*.

[19] Cheson BD, Horning SJ, Coiffier B (1999). Report of an international workshop to standardize response criteria for non-Hodgkin’s lymphomas. *Journal of Clinical Oncology*.

[20] Kaplan E, Meier P (1958). Nonparametric estimation from incomplete observations. *Journal of the American Statistical Association*.

[21] Cox D (1972). Regression models and life tables. *Journal of the Royal Statistical Society B*.

[22] Porrata LF, Inwards DJ, Lacy MQ, Markovic SN (2001). Immunomodulation of early engrafted natural killer cells with interleukin-2 and interferon-*α* in autologous stem cell transplantation. *Bone Marrow Transplantation*.

[23] Baiocchi G, Scambia G, Benedetti P (1993). Autologous stem cell transplantation: sequential production of hematopoietic cytokines underlying granulocyte recovery. *Cancer Research*.

[24] Guillaume T, Sekhavat M, Rubinstein DB, Hamdan O, Leblanc P, Symann ML (1994). Defective cytokine production following autologous stem cell transplantation for solid tumors and hematologic malignancies regardless of bone marrow or peripheral origin and lack of evidence for a role for interleukin-10 in delayed immune reconstitution. *Cancer Research*.

[25] Ma A, Koka R, Burkett P (2006). Diverse functions of IL-2, IL-15, and IL-7 in lymphoid homeostasis. *Annual Review of Immunology*.

[26] Carson WE, Fehniger TA, Haldar S (1997). A potential role for interleukin-15 in the regulation of human natural killer cell survival. *Journal of Clinical Investigation*.

[27] Boyiadzis M, Memon S, Carson J (2008). Up-regulation of NK cell activating receptors following allogeneic hematopoietic stem cell transplantation under a lymphodepleting reduced intensity regimen is associated with elevated IL-15 levels. *Biology of Blood and Marrow Transplantation*.

[28] Waldmann TA (2006). The biology of interleukin-2 and interleukin-15: implications for cancer therapy and vaccine design. *Nature Reviews Immunology*.

[29] Dean R, Masci P, Pohlman B (2005). Dendritic cells in autologous hematopoietic stem cell transplantation for diffuse large B-cell lymphoma: graft content and post transplant recovery predict survival. *Bone Marrow Transplantation*.

[30] Doherty TM, Seder RA, Sher A (1996). Induction and regulation of IL-15 expression in murine macrophages. *Journal of Immunology*.

[31] Neely GG, Robbins SM, Amankwah EK (2001). Lipopolysaccharide-stimulated or granulocyte-macrophage colony-stimulating factor-stimulated monocytes rapidly express biologically active IL-15 on their cell surface independent of new protein synthesis. *Journal of Immunology*.

[32] Joao C, Porrata LF, Burgstaler EA (2006). Immunologic autograft engineering by manipulation of apheresis machine collection settings. *Biology of Blood and Marrow Transplantation*.

